# Exosomes on the development and progression of renal fibrosis

**DOI:** 10.1111/cpr.13677

**Published:** 2024-06-19

**Authors:** Peihan Wang, Wu Chen, Bojun li, Songyuan Yang, Wei Li, Sheng Zhao, Jinzhuo Ning, Xiangjun Zhou, Fan Cheng

**Affiliations:** ^1^ Department of Urology Renmin Hospital of Wuhan University Wuhan Hubei P.R. China; ^2^ Department of Anesthesiology Renmin Hospital of Wuhan University Wuhan Hubei P.R. China

## Abstract

Renal fibrosis is a prevalent pathological alteration that occurs throughout the progression of primary and secondary renal disorders towards end‐stage renal disease. As a complex and irreversible pathophysiological phenomenon, it includes a sequence of intricate regulatory processes at the molecular and cellular levels. Exosomes are a distinct category of extracellular vesicles that play a crucial role in facilitating intercellular communication. Multiple pathways are regulated by exosomes produced by various cell types, including tubular epithelial cells and mesenchymal stem cells, in the context of renal fibrosis. Furthermore, research has shown that exosomes present in bodily fluids, including urine and blood, may be indicators of renal fibrosis. However, the regulatory mechanism of exosomes in renal fibrosis has not been fully elucidated. This article reviewed and analysed the various mechanisms by which exosomes regulate renal fibrosis, which may provide new ideas for further study of the pathophysiological process of renal fibrosis and targeted treatment of renal fibrosis with exosomes.

## Graphical abstract

Multiple pathways are regulated by exosomes produced by various cell types, including tubular epithelial cells and mesenchymal stem cells, in the context of renal fibrosis. The study of exosomes will explore their potential in translational medicine and provide new ideas and ways for creating effective clinical treatment strategies.

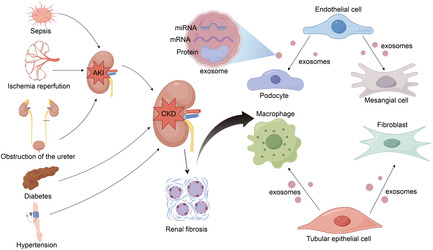

## 1 INTRODUCTION

Renal fibrosis is frequently outcome of chronic kidney disease (CKD) arising from several causes and is characterized by a sequence of pathological alterations, including the infiltration of inflammatory cells, proliferation of fibroblasts, and deposition of extracellular matrix.^1^ It has been reported that more than half of the adults over 70 years of age are affected by renal fibrosis.^2^ Sepsis, renal ischaemia–reperfusion, ureteral obstruction, and drug‐induced acute kidney injury (AKI) are often associated with the development of CKD. CKD may be an outcome of the deterioration of AKI. It is worth noting that chronic diseases associated with aging such as diabetes and hypertension are also important initiating factors of CKD. Because CKD is the terminal manifestation of various diseases related to renal function impairment, its incidence remains high.^3^, ^4^ However, there is no specific treatment for renal fibrosis in CKD, and dialysis replacement therapy for renal function damage caused by fibrosis has certain limitations.^5^, ^6^ The increasing prevalence of renal fibrosis resulting from CKD and the irreversible impairment of renal function associated with renal fibrosis have rendered it a significant public health concern with profound implications for human health. Therefore, it is essential to comprehensively elucidate the regulatory mechanisms underlying renal fibrosis at the molecular and cytological levels.^7^

Exosomes are extracellular vesicles that are generated by cells via a process known as budding. These vesicles may be created under both physiological and pathological conditions. Exosomes can encapsulate a diverse array of biological constituents, including messenger RNAs (mRNAs), small interfering RNAs, circular RNAs (circRNAs), proteins, lipids, and so on.^8^ In recent years, increasing evidence has shown that some exosomes have targeting properties, and their contents are functional and specific.^9^ The ability of exosomes to transmit information through extracellular vesicles has been called the third mode of intercellular communication, in addition to cell signal transmission through contact or soluble molecules.^10^ Exosomes mediate cell communication and play a unique role in maintaining intracellular environmental homeostasis by regulating cell physiological death, cell inflammation and cell differentiation, and they are closely related to many aspects of human health and disease.^11^ For example, exosomes can regulate the inflammatory response in cells induced by various factors by promoting or inhibiting inflammasome activation.^12^ Exosomes have garnered significant attention as a burgeoning area of study due to their clinical use in illness detection and therapy.^13^, ^14^

**FIGURE 1:**
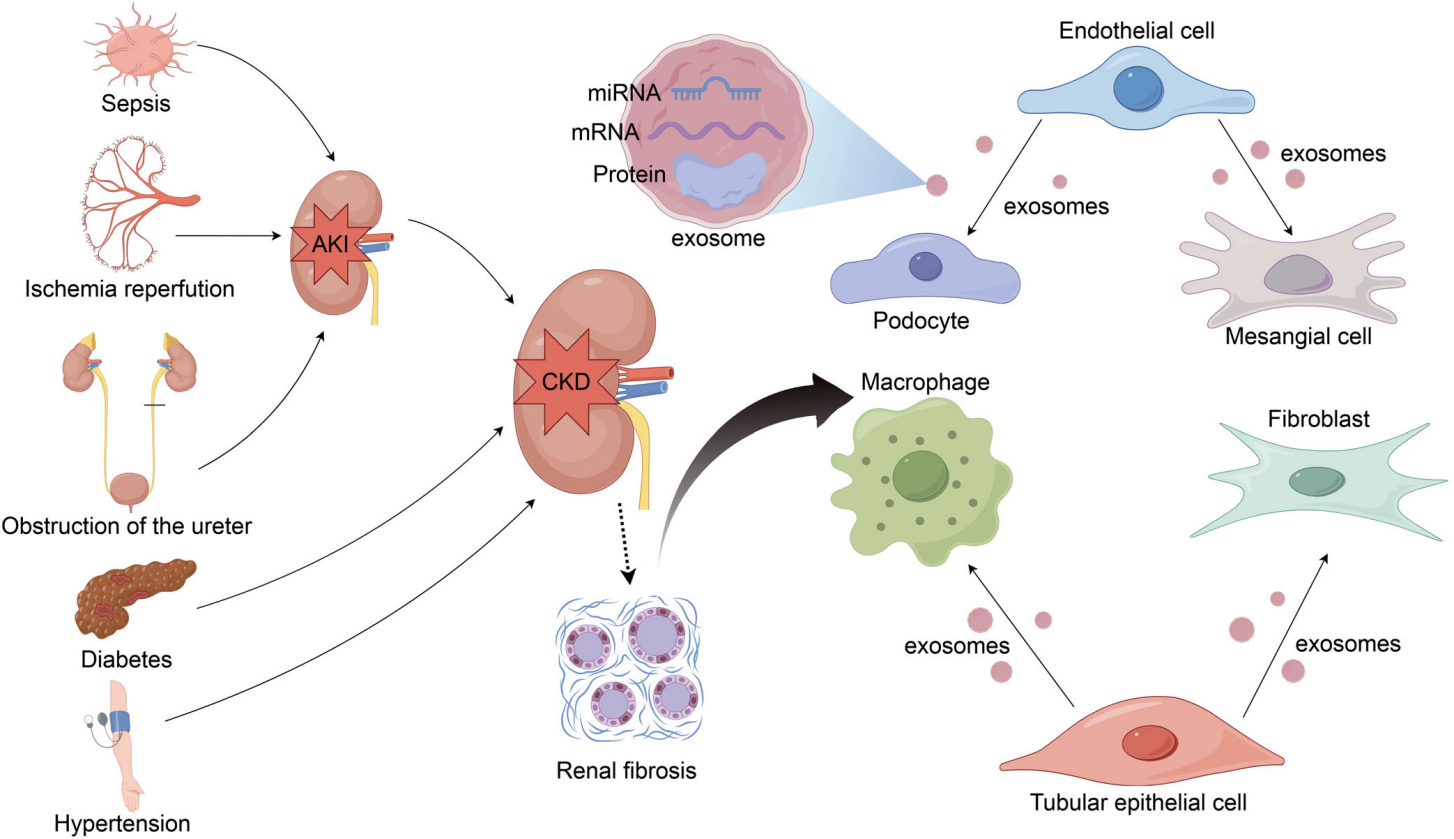
Sepsis, ischaemia–reperfusion, ureteral obstruction, and other factors can lead to acute kidney injury (AKI). AKI can progress to chronic kidney disease (CKD), and factors such as diabetes and hypertension can also lead to CKD. Renal fibrosis is a pathological process in CKD. Renal TECs can release exosomes to macrophages and fibroblasts, and endothelial cells can release exosomes to podocytes and glomerular mesangial cells. Exosomes mediate information transfer and cargo transport between these cells. Exosomes play a role in the process of renal fibrosis by transporting microRNAs (miRNAs), mRNAs, proteins, and so on.

Numerous studies have shown that exosomes derived from a variety of cells, such as renal tubular epithelial cells (TECs) and macrophages, play a unique role in maintaining normal physiological function in the kidney.^15^ In addition, there is evidence that exosomes in a variety of bodily fluids, such as blood, urine, and saliva, can be used as biomarkers for tumours, atherosclerosis, and a variety of kidney diseases and have the potential to assist in disease diagnosis and targeted therapy.^16^, ^17^, ^18^ With further study of the regulatory mechanism of renal fibrosis, which is a complex pathophysiological process, the role of exosomes and their contents in renal fibrosis has gradually gained attention (Figure 1). Clarifying the mechanism by which exosomes regulate renal fibrosis may provide new ideas for the treatment of patients with renal fibrosis and end‐stage CKD. This review focuses on the regulatory role of exosomes derived from various sources in renal fibrosis to find therapeutic targets and strategies for renal fibrosis.

## 2 THE RELEASE AND UPTAKE OF EXOSOMES

**FIGURE 2:**
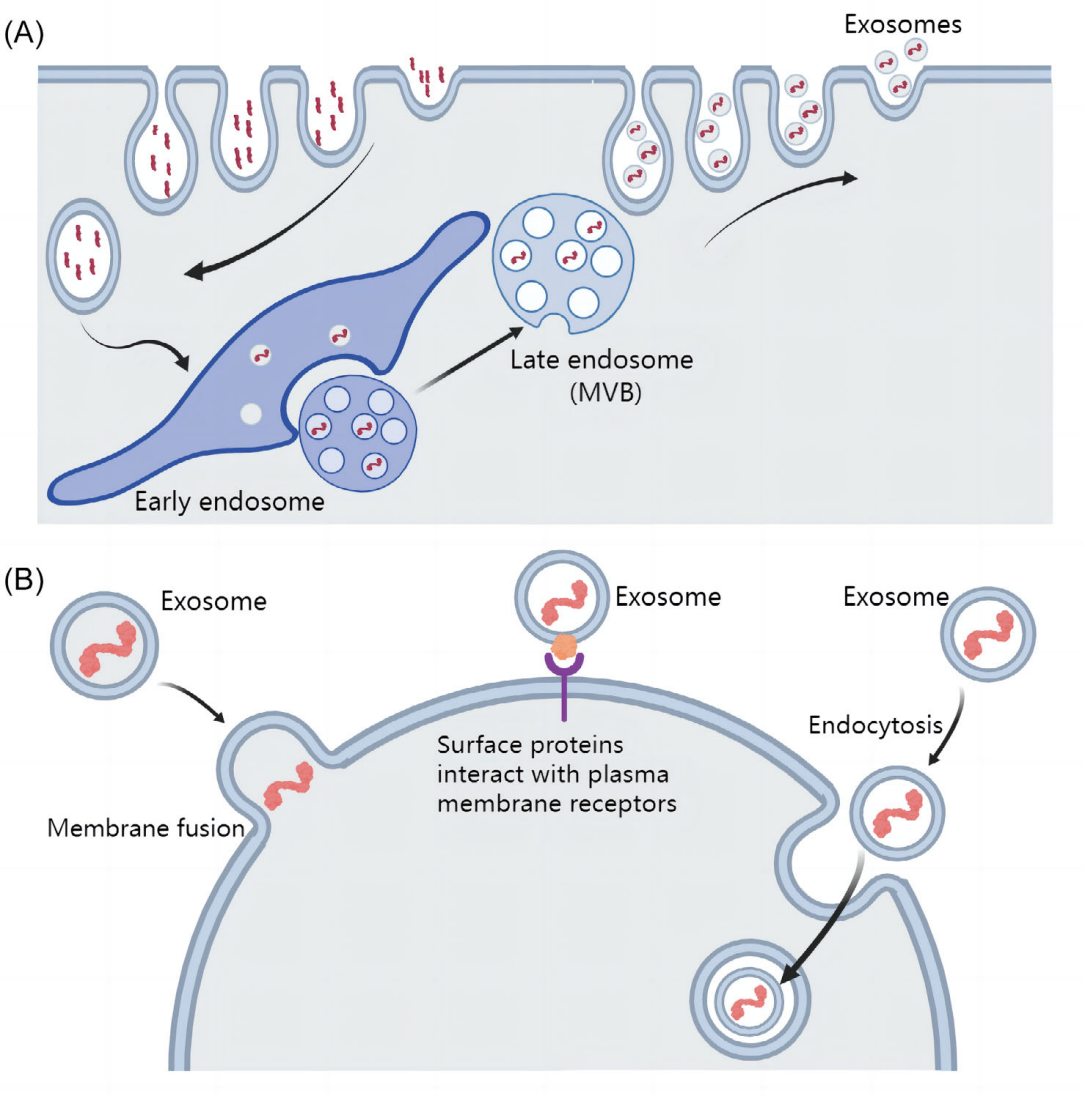
Release and uptake of exosomes. (A) Molecular cargo enters the cell by endocytosis and is subsequently sorted, clustered together, and forms early endosomes. Part of the early endosomes is lysed by lysosomes, while the other part forms late endosomes containing a large number of vesicles, which eventually escape from the cell through exocytosis. These vesicles are called exosomes. (B) There are several ways for exosomes to be taken up by target cells: the fusion of the exosome membrane with the cell membrane to release molecular cargo into the cell, and the endocytosis of the cell to exosomes. In addition, some exosomes do not need to enter cells, and their surface proteins bind to target cell surface ligand proteins to play a role in information transmission.

Exosomes, formed by the fusion of a subset of late endosomes, are a class of vesicles that are released into the extracellular space by the cytoplasmic membrane.^8^, ^19^ Specifically, the cell membrane wraps and transports various molecular cargo into initial vesicles, called early endosomes, by “budding.” Subsequently, cargo not recycled by the cell migrates towards the vacuolar regions of early endosomes and form late endosomes. Some late endosomes are degraded by lysosomes, while others, called multivesicular bodies, migrate to the cell membrane and are finally released to the extracellular space, where they are called exosomes.^11^, ^15^, ^20^ To regulate the function of target cells, exosomes must first be taken up by target cells. At present, there are two main ways of cellular uptake of exosomes, namely membrane fusion and endocytosis. Endocytosis can be divided into phagocytosis, microphagocytosis, endocytosis mediated by lipid rafts, clathrins, or fossa proteins.^21^, ^22^ Exosomes are taken up by target cells to transport cargo molecules to target cells, thus exerting their function of mediating intercellular communication. In addition, it is worth noting that there are also exosome surface proteins that can directly bind to receptor proteins on the surface of the plasma membrane of specific target cells to mediate information transfer, and in this mode, exosomes do not need to be taken up by target cells (Figure 2).^23^

## 3 THE ROLE OF EXOSOMES IN RENAL PHYSIOLOGY

Exosomes play an important role in kidney development and regeneration. Previous studies have shown that exosomes mediate complex signal transduction between the ureteric bud and the metanephric mesenchyme during kidney development, such by activating the Wnt pathway, which is indispensable for kidney development.^24^ In addition, renal TEC‐derived exosomes have been shown to induce mesenchymal‐to‐epithelial transition in the kidney, thereby transforming the mesenchyme into a renal tubular epithelial structure, and mesenchymal cell‐derived exosomes can inhibit epithelial–mesenchymal transition (EMT) in HK‐2 cells.^25^, ^26^ In addition to their important roles in kidney development and regeneration, exosomes also play roles in clearance, material delivery, signal communication, and immune and inflammatory regulation in the nephron.

In recent years, a significant amount of research has been conducted on the role of exosomes in renal physiological function and the underlying molecular mechanisms. Numerous studies have shown that macrophages, fibroblasts, T lymphocytes, monocytes and natural killer cells can communicate with TECs through exosomes, thereby affecting the normal physiological function of the kidney and pathological changes such as AKI, renal cancer, and renal fibrosis.^27^, ^28^ The maintenance of normal physiological function of the kidney is inseparable from signal transduction and the transmission of material between different segments of the nephron. Exosomes can be transferred from the proximal tubule to the distal convoluted tubule and collecting duct to mediate information transmission and perform a series of physiological functions, such as reducing the basal reactive oxygen species (ROS) production rate of recipient cells.^29^ Another study showed that exosomes derived from collecting duct cells could induce the expression of aquaporin 2 (AQP‐2) in recipient cells.^30^ It is well known that AQP‐2 is present in renal collecting duct cells, and so exosomes play a role in renal signalling to regulate the reabsorption of water by the kidney.^31^

In addition to functions related to cell communication, such as signal transduction, receptor transfer, and miRNA transport, exosomes in the kidney maintain normal cellular activity by transporting toxic substances out of the cell.^15^, ^32^ For example, caspase‐3 is an apoptotic terminal cleavage enzyme that plays an indispensable role in apoptosis and mediates gasdermin‐E‐induced pyroptosis.^33^, ^34^ In the early stage of apoptosis, renal cells can transport intracellular caspase‐3 out of the cell through exosomes to promote cell survival and the compensatory proliferation of recipient cells.^35^

Immune cells such as lymphocytes and macrophages can release exosomes to affect TECs, and renal TEC‐derived exosomes can act on immune cells. As a communication mechanism between immune cells and TECs, exosomes play an important role in the regulation of the renal inflammatory response.^36^, ^37^ For example, Shen et al. found that exosomes derived from Adipose‐derived stem cells could reduce the accumulation of ROS in macrophages and the release of inflammatory factors such as IL‐1β, TNF‐α, and IL‐6 by regulating the Nrf2/HO‐1 axis, thereby reducing inflammation.^38^ The role of exosomes in regulating inflammation in the kidney may be related to regulating the phenotypic transformation of recipient cells (macrophages, T lymphocytes, etc.) via miRNAs and immunomodulatory proteins released by exosomes.^39^, ^40^ In summary, exosomes play an important role in maintaining the normal physiological function of the kidney by selectively packaging cargo and transporting it to target cells, which take up this cargo. Understanding the role of exosomes in renal physiology may provide a theoretical basis for subsequent research.

## 4 THE REGULATION OF RENAL FIBROSIS BY EXOSOMES

### 4.1 Exosomes regulate renal fibrosis by regulating EMT in TECs

EMT is a multifaceted biological process characterized by the conversion of epithelial cells into mesenchymal cells, which acquire distinct phenotypic traits. This process plays a significant role in cellular plasticity and is implicated in several physiological and pathological states.^41^, ^42^ EMT is important in controlling inflammation, tissue healing, cancer formation, and fibrosis in many organs and tissues.^43^, ^44^ The signalling pathway regulated by TGF‐β affects the induction of several distinctive characteristics of EMT. These characteristics include the management of genes linked to the extracellular matrix, the control of cell–cell interactions, modification of the actin‐based cytoskeleton, and the regulation of additional growth factors and cytokines.^45^, ^46^ Numerous studies have shown that excessive activation of the TGF‐β signalling pathway can promote EMT, extracellular matrix deposition and fibroblast formation.^47^ The transformation of specialized epithelial cells into profibrotic and pro‐inflammatory myofibroblasts is a major form of EMT that is closely associated with organ fibrosis. Myofibroblasts produce stiff scars that are rich in collagen, destroy tissue structure, change the biochemical and biophysical microenvironment, and lead to tissue fibrosis and abnormalities.^48^, ^49^ Andugulapati et al. found that TGF‐β‐mediated EMT promoted collagen deposition and the progression of pulmonary fibrosis.^50^ Pan et al. found that activation of the TGF‐β1/smad pathway promoted EMT and liver fibrosis in liver epithelial cells.^51^ Similarly, overactivation of EMT in TECs and glomerular endothelial cells promoted renal fibrosis.^52^, ^53^, ^54^

**FIGURE 3:**
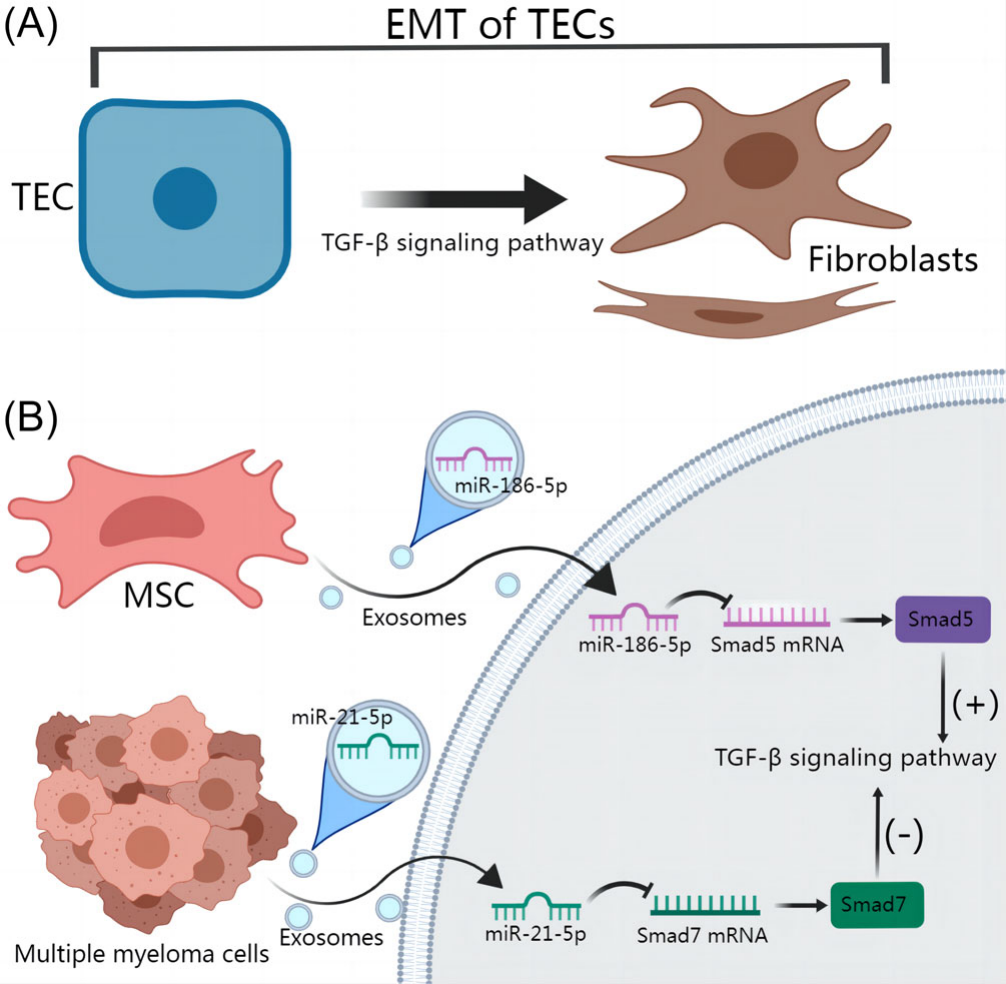
Exosomes regulate renal fibrosis by regulating epithelial–mesenchymal transition (EMT) in tubular epithelial cells (TECs). (A) TECs can transform into fibroblasts through EMT, which is closely related to TGF‐β signalling pathway. (B) Exosomal miR‐186‐5p derived from mesenchymal stem cells (MSCs) targeted and inhibited Smad5, which is an activator of TGF‐β signalling pathway, in TECs. Finally, EMT was inhibited. Exosomal miR‐21‐5p derived from Multiple myeloma cells targeted and inhibited Smad7, which is an inhibitor of TGF‐β signalling pathway, in TECs. Finally, EMT was promoted.

Nagaishi et al. first discovered that the administration of bone marrow mesenchymal stem cells (MSCs) improved renal fibrosis in a mouse model of diabetic nephropathy. This improvement was attributed to the secretion of exosomes by MSCs. The underlying mechanism involved the downregulation of TGF‐β1 expression, which subsequently inhibited EMT in TECs.^55^ This work represents a preliminary investigation into the therapeutic potential of exosomes derived from MSCs for the treatment of renal fibrosis. However, the specific molecular components involved in the effects of these exosomes have not been explored in depth. Zhu et al. then examined adipose‐derived mesenchymal stem cells (ADMscs) and found that ADMscs prevented TGF‐β1‐induced profibrotic phenotypic transformation in TECs.^56^ These studies indicate that the administration of exosomes produced by MSCs may be a viable therapeutic strategy for the treatment of renal fibrosis. Furthermore, a study conducted in 2018 revealed that measuring miR‐29c levels in urine exosomes could be a diagnostic marker for renal interstitial fibrosis. This marker has been implicated in the promotion of EMT in TECs. However, the precise mechanism underlying this phenomenon remains unexplored.^57^ In 2020, researchers found that miR‐29c reduced EMT and renal fibrosis through the TPM1‐mediated Wnt/β‐catenin pathway.^58^ Therefore, it is reasonable to speculate that exosomes can carry miR‐29c to regulate the TPM1‐mediated Wnt/β‐catenin pathway, thereby regulating EMT and renal fibrosis, but this topic needs to be further studied. Moreover, MSC‐derived exosomes were used by Yang et al. to deliver miR‐186‐5p to a normal rat proximal TEC line or mouse kidneys in vitro and in vivo. MSC‐derived exosomes delivered miR‐186‐5p to reduce EMT in TECs by targeting Smad5.^59^ Similarly, many studies have shown that MSCs communicate with TECs through exosomes; in other words, exosomes attenuate EMT of TECs and alleviate renal fibrosis by transporting miRNA and other substances.^26^, ^60^, ^61^ In contrast to the inhibitory effects of exosomes derived from MSCs on renal EMT, exosomal miR‐21‐5p derived from multiple myeloma cells could enhance renal EMT by selectively targeting the TGF‐β/SMAD7 signalling pathway (Figure 3).^62^ The literature mostly focuses on investigating the influence of MSCs on EMT in TECs through exosomes. However, little attention has been given to the effect of exosomes derived from TECs, macrophages, and other cellular sources on the regulation of renal fibrosis via EMT. We believe that the latter is a direction worth exploring.

### 4.2 Exosomes regulate renal fibrosis by regulating EMT in glomerular endothelial cells

Glomerular endothelial cells are an integral cellular component of the glomerulus and play a crucial role in forming the inner wall of the glomerular filtration barrier inside the nephron. Endothelial cell impairment often has a significant effect on renal function.^63^ Studies have shown that exosomes can mediate sepsis‐induced endothelial cell dysfunction, but the specific mechanism is unknown.^64^ Endothelial–mesenchymal transition (EndMT) is an important factor in the functional changes and damage to endothelial cells, which is a complex process involving the transformation of endothelial cells into mesenchymal cells such as myofibroblasts.^65^ This process involves alterations in gene or protein expression, such as the loss of endothelial markers such as VE‐cadherin and CD31, and gain of mesenchymal or myofibroblast markers such as SM22α and α‐smooth muscle actin (α‐SMA).^66^ These proteins or genes may be used as indicators of EndMT in experiments. Interestingly, the regulatory pathway of EndMT seems to be inseparable from TGF‐β, and the regulation of EndMT has some similarities with the regulation of EMT.^67^ In addition, it has been shown that the Hippo pathway is involved in the induction of EndMT.^68^ Some studies have shown that autophagy‐related pathways can affect the occurrence of EndMT; for example, Dong et al. showed that autophagy mediated by the PI3K/AKT/mTOR pathway could alleviate the occurrence of EndMT.^69^ EndMT‐mediated fibrosis in tissues and organs has a significant effect on the development of many illnesses, including atherosclerosis, haemangioma, diabetes, pulmonary fibrosis, liver cirrhosis, and renal fibrosis.^70^, ^71^, ^72^, ^73^, ^74^

**FIGURE 4:**
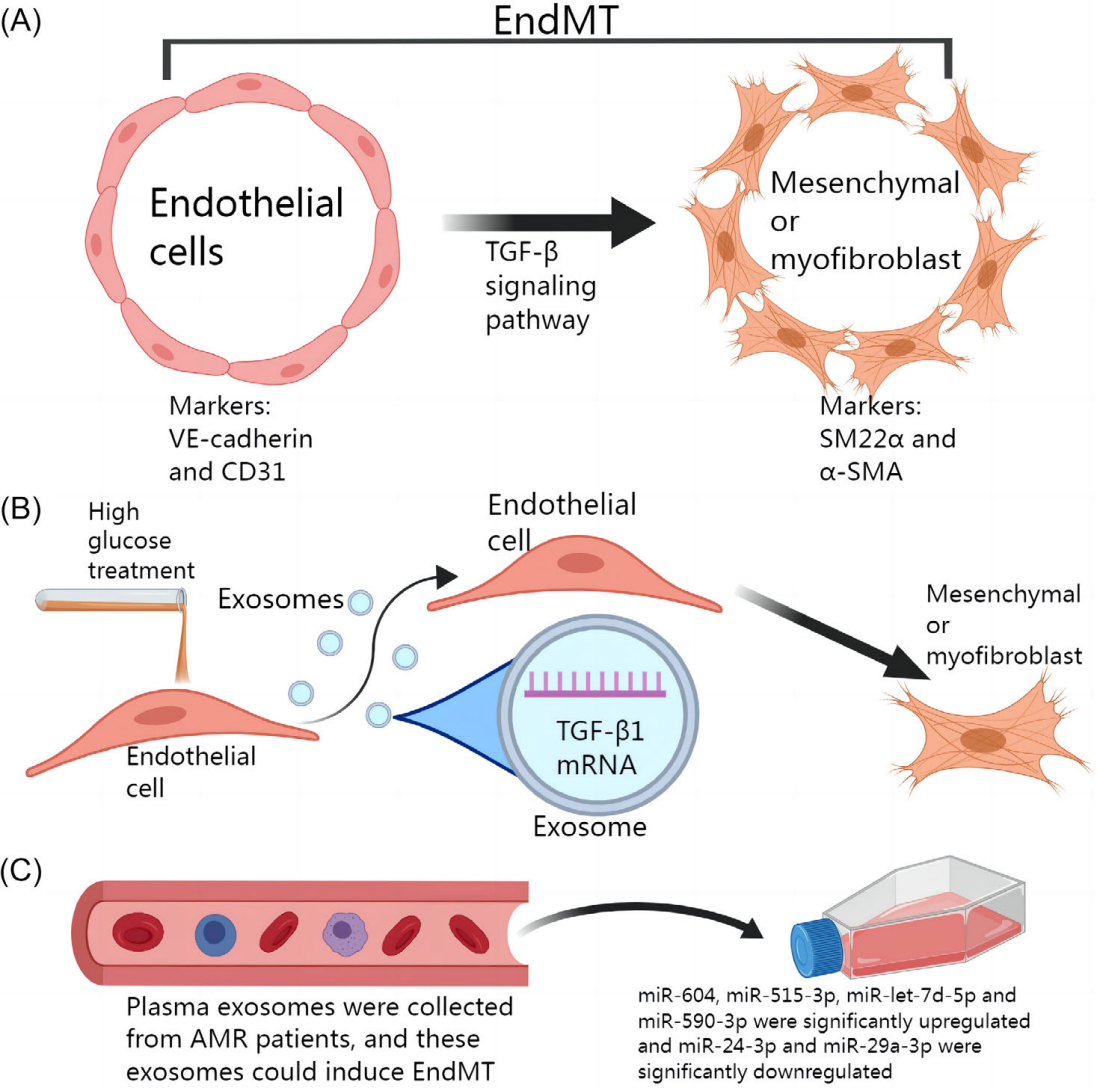
Exosomes regulate renal fibrosis by regulating endothelial–mesenchymal transition (EndMT) in glomerular endothelial cells. (A) Endothelial cells can transform into mesenchymal cells or fibroblasts, a process known as EndMT. The markers of endothelial cells were VE‐cadherin and CD31, and the markers of mesenchymal or myofibroblasts were SM22α and α‐SMA. (B) Glomerular endothelial cells treated with high glucose can transport exosomes rich in TGF‐β1 mRNA to neighbouring cells to induce EndMT. (C) Exosomes derived from endothelial cells in the plasma of AMR patients have been shown to induce EndMT. In these exosomes, miR‐604, miR‐515‐3p, miR‐let‐7d‐5p, and miR‐590‐3p were significantly upregulated, while miR‐24‐3p and miR‐29a‐3p were significantly downregulated.

Blocking EndMT reduced inflammation and fibrosis in a mouse model of renal fibrosis induced by unilateral ureteral obstruction and folic acid.^75^ In addition, the transformation of endothelial cells into fibroblasts can be observed in high glucose‐induced diabetic nephropathy models. Interestingly, high glucose treatment caused endothelial cells to release more exosomes, and TGF‐β1 mRNA was confirmed to be enriched in these exosomes.^76^ Furthermore, TGF‐β has been shown to induce EndMT in glomerular endothelial cells. Exosomes can mediate signal transduction between endothelial cells and activate TGF‐β1‐mediated EndMT.^67^ A recent study provided strong evidence that exosomes regulated EndMT‐induced renal fibrosis.^77^ Severe renal fibrosis can occur after kidney transplantation, which can be attributed in part to antibody‐mediated rejection.^78^, ^79^ Flow cytometric analysis of exosomes in the plasma of AMR patients revealed that many exosomes were derived from endothelial cells. These exosomes have been confirmed to induce EndMT. In addition, miR‐604, miR‐515‐3p, miR‐let‐7d‐5p, and miR‐590‐3p were significantly upregulated and miR‐24‐3p and miR‐29a‐3p were significantly downregulated in exosomes in the AMR group compared with the transplantation control group.^77^ These studies indicate that exosomes can promote renal fibrosis by transporting related miRNAs and TGF‐β1 mRNA to induce EndMT, which plays a positive role in the pathogenesis of diseases such as diabetic nephropathy and posttransplantation kidney injury (Figure 4).

### 4.3 Exosomes regulate renal fibrosis by regulating autophagy

Autophagy is a multifaceted biological process in which cytoplasmic proteins or organelles are sequestered and then transferred to lysosomes for destruction. The phenomenon in question is extensively observed in the metabolic processes of eukaryotic cells. It pertains to the quality control of cellular metabolism and serves a crucial role in the maintenance of homeostasis.^80^ Autophagy is observed in several types of renal cells, such as TECs, podocytes, glomerular endothelial cells, and mesangial cells. At the molecular and cellular levels, autophagy plays a crucial role in regulating the clearance of hazardous chemicals and the stress response in renal cells, ensuring the maintenance of intracellular homeostasis.^81^ Autophagy plays a protective role in kidney injury; however, reports have shown that autophagy can aggravate kidney injury in certain ways. Some researchers believe that autophagy is involved in the repair of damaged cells during the transition from AKI to CKD. In general, autophagy plays a protective role in the kidney and promotes renal fibrosis.^82^ For example, Livingston et al. showed that after AKI, TECs release large amounts of fibroblast growth factor 2, which depends on the activation of autophagy, thereby promoting renal fibrosis.^83^ However, studies have shown that the activation of autophagy can reduce renal interstitial fibrosis.^84^, ^85^, ^86^ Therefore, the effect of autophagy on renal fibrosis is complex. In addition, autophagy interacts with cell apoptosis, EMT and other processes, and these cellular processes affect renal fibrosis in different ways, making it difficult to further study the effect of exosome‐regulated autophagy on renal fibrosis.^69^, ^80^

**FIGURE 5:**
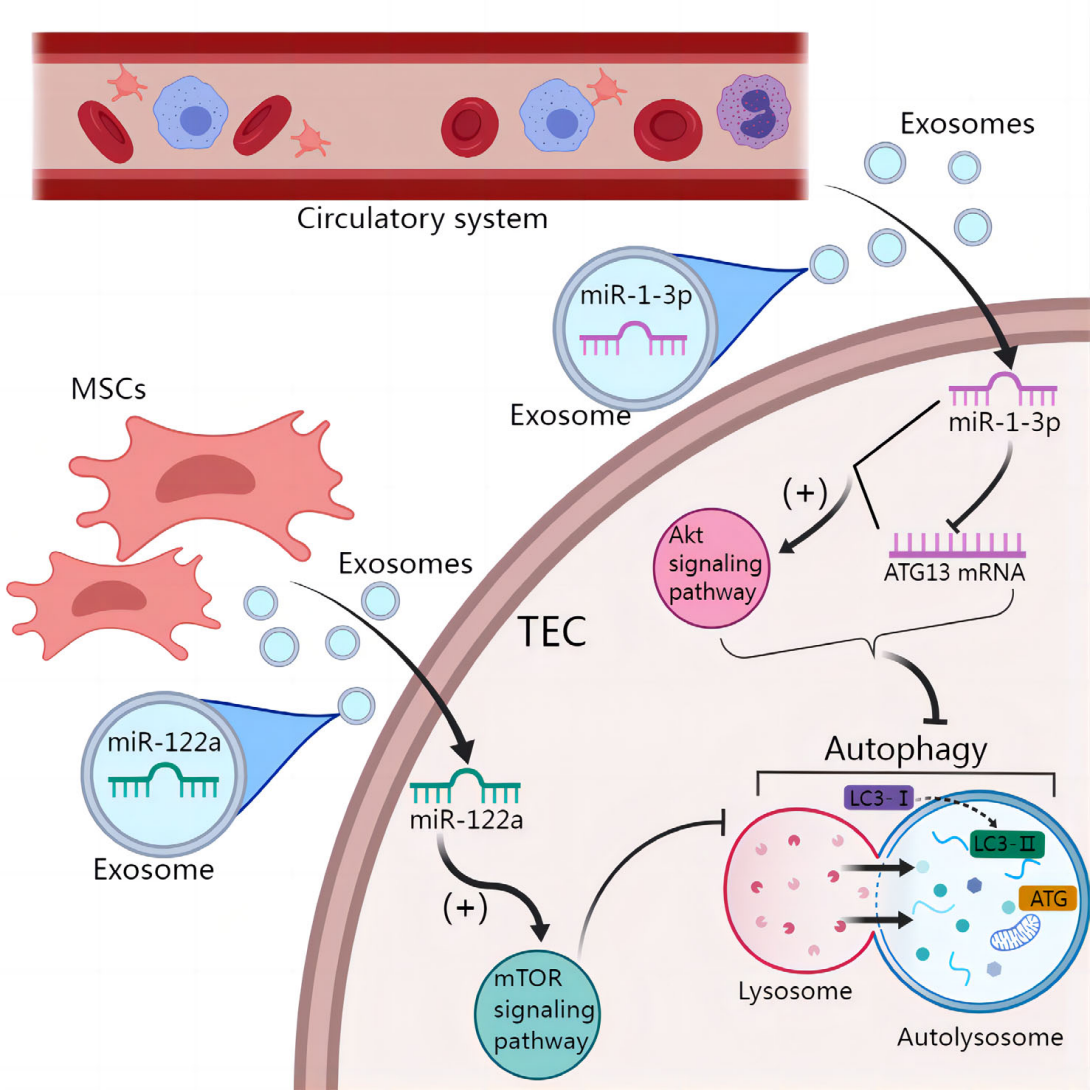
Exosomes regulate renal fibrosis by regulating autophagy. Circulating exosomes delivered miR‐1‐3p to TECs, miR‐1‐13p targeted and inhibited the expression of ATG13, activated Akt signalling pathway, and inhibited autophagy. Mesenchymal stem cells (MSCs)‐derived exosomal miR‐122a can activate mTOR‐signalling pathway to inhibit autophagy. During autophagy, cytoplasmic LC3‐I‐catalyses the enzymolysis of a small fragment of polypeptide, which was followed by conversion to membrane‐type LC3‐II.

Autophagy involves the enzymatic cleavage of a small portion of the cytoplasmic LC3‐I protein, resulting in the conversion of LC3‐I to LC3‐II. Moreover, Beclin1 is a protein that has a positive regulatory effect on autophagy via its interaction and binding with the autophagy‐related protein (ATG) ATG14. Hence, the assessment of autophagy levels may be accomplished by identifying autophagy‐associated proteins, such as LC3‐II and Beclin1, and evaluating their expression.^87^, ^88^ In addition, many studies have shown that exosomes regulate autophagy levels by altering the mTOR signalling pathway, which is an autophagy inhibitory pathway.^89^ Although exosomes can regulate autophagy in a variety of ways, many studies have focused on exosome‐mediated inhibition of the expression of their downstream target genes by transporting miRNAs, thereby regulating the activation or inhibition of autophagy‐related pathways.^90^ In 2018, Ebrahim et al. explored the mechanism by which MSC‐derived exosomes protect against diabetic nephropathy. In their study, mTOR mRNA and protein levels were significantly upregulated in mice with diabetic nephropathy and were significantly reduced by the infusion of MSC‐derived exosomes into the mice. LC3‐II was examined, and Beclin1 expressed by MSCs can inhibit the mTOR pathway, which promotes renal TEC autophagy and improves renal fibrosis caused by diabetic nephropathy.^91^ Although this study did not specifically determine which substances MSC exosomes deliver to target cells or explain how autophagy improves renal fibrosis, it provides a new idea for subsequent studies on how exosomes affect renal fibrosis by regulating autophagy. In 2021, a report showed that myocardial infarction increased circulating exosomes in the blood and their transfer to the kidney. In vitro experiments showed that the level of exosomal miR‐1‐3p was significantly upregulated, and miR‐1‐3p directly targeted the expression of the ATG ATG13, activated the AKT signalling pathway and inhibited autophagy in rat TECs, thereby reducing contrast‐induced kidney injury and reducing renal fibrosis.^92^ This study identified a specific exosome‐transported miRNA that regulated autophagy. Interestingly, in this study, autophagy promoted kidney injury. Overall, autophagy seems to be a double‐edged sword that can protect cells under stress conditions, while excessive activation of autophagy can induce cell death. Finally, a recent study showed that MSC‐derived exosomes could alleviate TGF‐β‐induced fibrosis in HK‐2 cells by regulating the mTOR signalling pathway and inhibiting downstream autophagy.^93^ In summary, exosomes can affect the level of autophagy by targeting downstream autophagy‐related regulatory pathways, such as mTOR or ATGs, thereby promoting or inhibiting renal fibrosis (Figure 5).

### 4.4 Exosomes regulate renal fibrosis by regulating macrophage polarization

Macrophages are a subset of innate immune cells characterized by their ability to perform phagocytosis. With further investigation of macrophages, increasing evidence indicates that these cells participate in the inflammatory response through complex and diverse processes and have unique regulatory effects on tissue repair and fibrosis.^94^ Macrophages often undergo phenotypic modification, which is known as polarization, as a result of disturbances in the microenvironment. Polarized macrophages can be broadly classified into two primary groups: classically activated M1 macrophages and alternatively activated M2 macrophages.^95^ M1 macrophages can be induced by lipopolysaccharide (LPS) alone or in combination with other cytokines, such as IFN‐γ and TNF‐α. These cells are characterized by the secretion of many inflammatory factors [IL‐12, IL‐1β, IL‐6, IL‐23, and TNF‐α]. M2 macrophages can be activated by IL‐4, IL‐13, IL‐10, and TGF‐β. M2 macrophages have anti‐inflammatory effects and mediate angiogenesis and tissue repair, while M1 macrophages have pro‐inflammatory functions in kidney disease. In addition, M2 macrophages can reduce inflammation and repair damaged tissue, but excessive repair is also a cause of fibrosis.^96^, ^97^, ^98^ Excessive polarization of M2 macrophages promotes renal fibrosis after AKI because M2 macrophages continue to infiltrate kidney tissues through the release of TNF‐β, platelet‐derived growth factor, and vascular endothelial growth factor, promoting the activation of fibroblasts.^99^, ^100^

**FIGURE 6:**
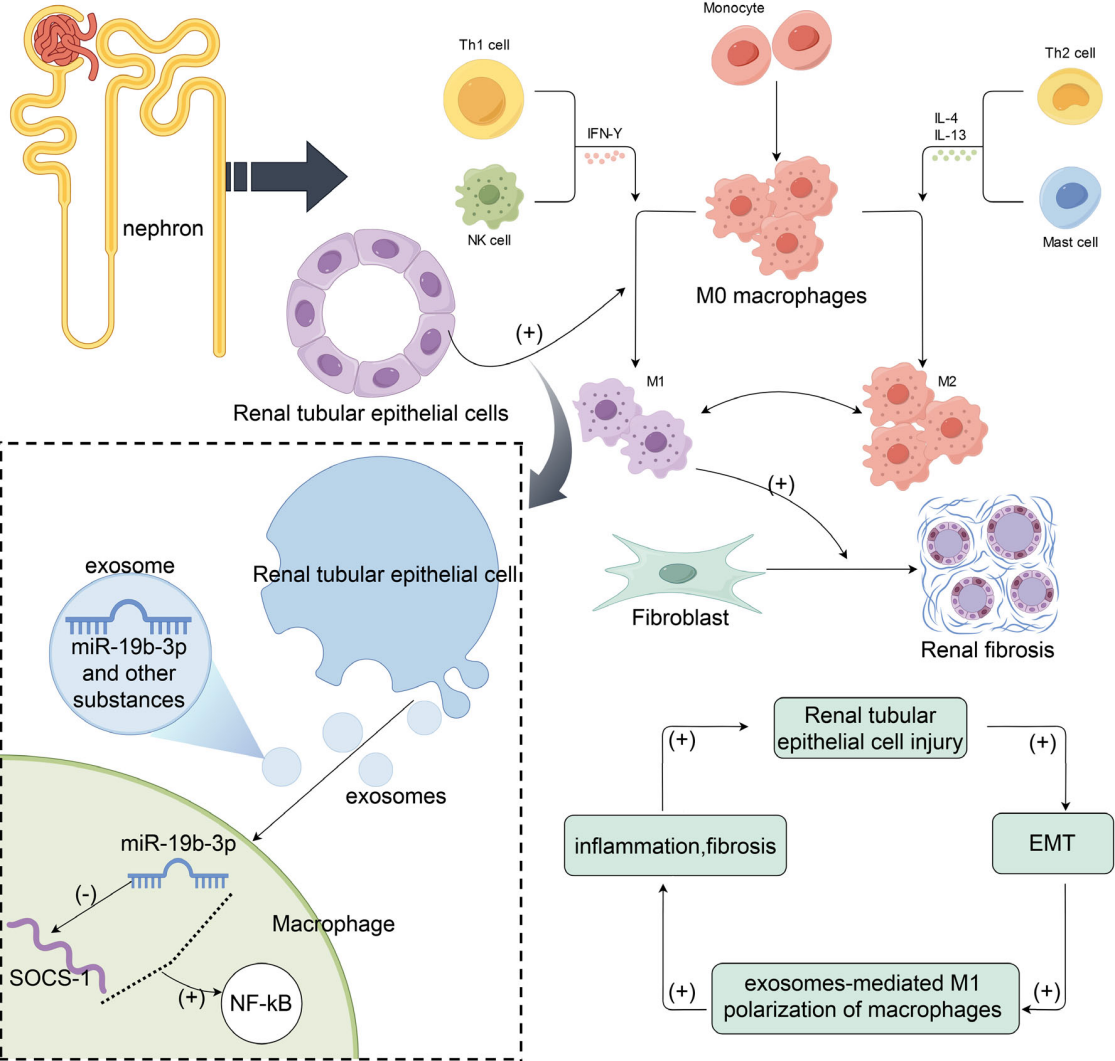
The basic process of macrophage polarization and the process of renal tubular cell‐derived exosomes promoting renal fibrosis by regulating M1 macrophage polarization. Specific known regulatory mechanisms are shown in parentheses. Tubular epithelial cells (TECs) secreted exosomes rich in miR‐19b‐3p after injury, and exosomes with high expression of miR‐19b‐3p entered macrophages. miR‐19b‐3p targeted and inhibited suppressor of cytokine signalling 1 (SOCS‐1), thereby activating the NF‐Κb signalling pathway and promoting the polarization of M1 macrophages, thereby aggravating the inflammatory response in the kidney. Renal TEC injury, epithelial–mesenchymal transition (EMT), exosomes‐mediated M1 macrophage polarization, inflammation, and fibrosis form a positive feedback loop.

In 2022, Lu et al. extracted and isolated exosomes derived from human bone marrow MSCs and found that these exosomes could alleviate renal fibrosis induced by hydronephrosis. Mechanistically, exosomes derived from MSCs (MSC‐Exos) inhibited M1 macrophage polarization.^101^ Another study in the same year used an ischaemia–reperfusion model to investigate renal fibrosis after AKI and found that MSC‐Exos significantly limited renal fibrosis after ischaemia–reperfusion injury. Interestingly, researchers found that MSCs‐Exo could significantly promote M2 macrophage polarization. A certain degree of M2 macrophage polarization inhibits inflammation and accelerates the repair process after kidney injury, thereby alleviating renal fibrosis.^94^, ^102^ Recently, it has been reported that renal TEC‐derived exosomes promote the release of inflammatory factors by regulating the polarization of M1 macrophages in the fibrotic kidney, which further aggravates kidney injury and fibrosis.^103^ This study suggests that there may be a positive feedback loop during renal fibrosis, and exosomes released by TECs undergoing EMT act on macrophages to promote the polarization of macrophages to the M1 phenotype, and the infiltration of M1 macrophages into kidney tissue will in turn aggravate inflammation and fibrosis. Finally, there was a report that TECs secrete exosomes containing miR‐19b‐3p after injury, thereby communicating macrophages, which highly express miR‐19b‐3p and target suppressor of cytokine signalling 1 (SOCS‐1) to enhance NF‐Κb activity. This process promotes the activation of M1 macrophages and aggravates the inflammatory response in the kidney.^104^ Inflammation plays a key role in the occurrence and development of renal fibrosis. Studies have shown that the infiltration of pro‐inflammatory M1 macrophages mediates renal inflammation to aggravate renal injury, and persistent M1‐dependent renal injury eventually leads to fibrosis.^105^, ^106^ In conclusion, exosomes can mediate signal transduction and the polarization of macrophages, thereby affecting the progression of renal interstitial fibrosis. Macrophage polarization is closely related to the status of TECs. Exosomes derived from TECs are involved in the interaction between macrophages and TECs and regulate renal fibrosis (Figure 6).

### 4.5 Exosomes regulate renal fibrosis by regulating fibroblast activation

Renal interstitial fibroblasts, which are located in the renal capsule and renal tubular basement membrane, possess a diverse range of biological functions. The intimate association between the proliferation and activation of fibroblasts and the deposition of extracellular matrix underscores the significant role of fibroblasts in the development of renal fibrosis under pathological conditions.^107^ During the progression of renal fibrosis, damage first occurs to TECs, followed by the subsequent proliferation and activation of fibroblasts. There are empirical data supporting the presence of crosstalk between TECs and renal interstitial fibroblasts, which plays a role in renal injury and healing, as well as the regulation of fibrosis development. The interaction between TECs and renal interstitial fibroblasts is facilitated by the release and uptake of exosomes.^108^, ^109^ Therefore, renal TEC‐derived exosomes may participate in the epigenetic regulation of renal interstitial fibroblast activation by transporting genetic material or proteins. Clarifying the mechanism of this process can elucidate the in‐depth mechanism of renal fibrosis and guide treatment.

**FIGURE 7:**
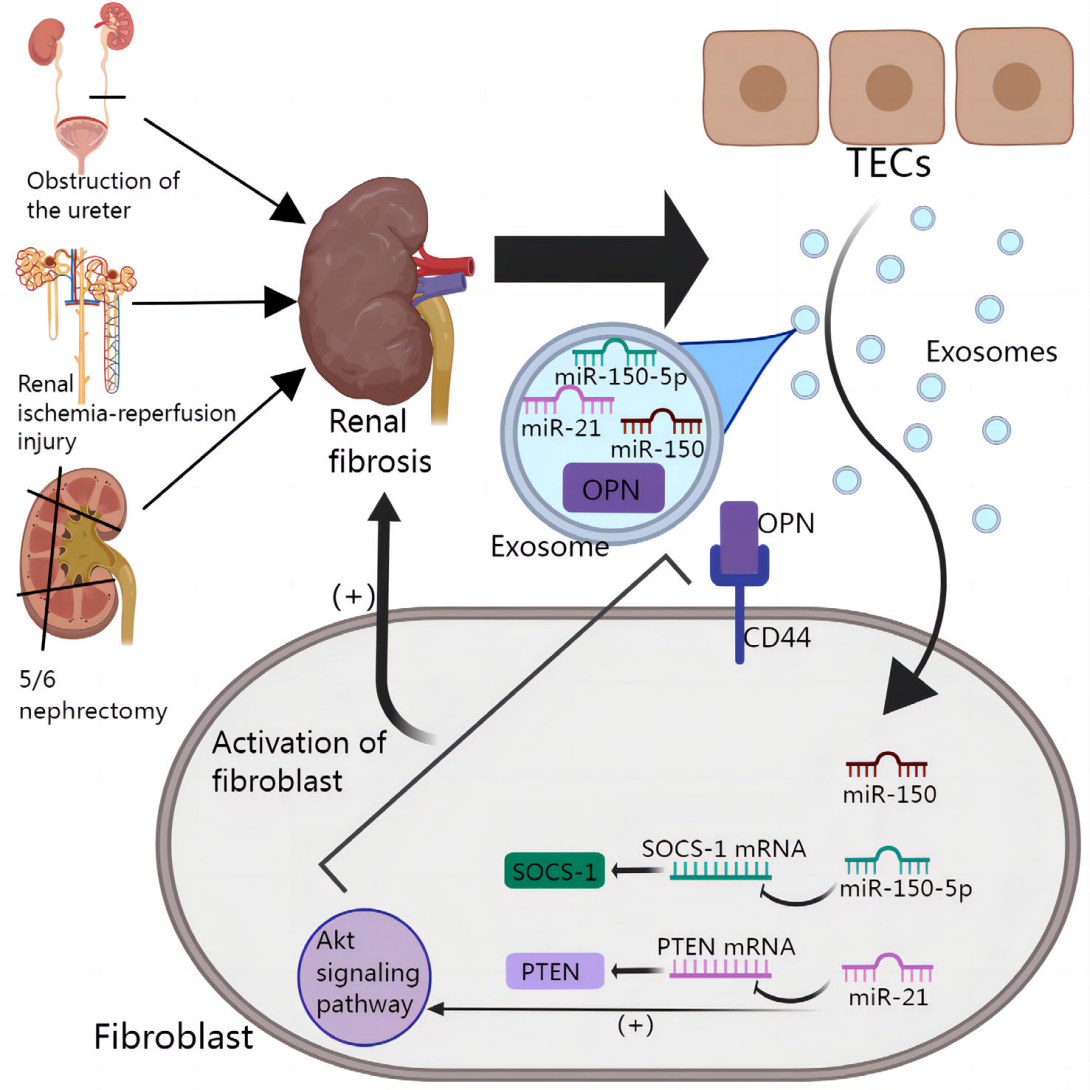
Exosomes regulate renal fibrosis by regulating fibroblast activation. TECs release more exosomes in models of unilateral ureteral obstruction, renal ischaemia–reperfusion injury (RIRI), or 5/6 nephrectomy. These exosomes are enriched in OPN, Mir‐150‐5p, miR‐21, and miR‐150, and exosomes activate fibroblasts by transporting these molecular cargo to fibroblasts. Concretely, miR‐150‐5p targets SOC‐1; miR‐21 targets PTEN and activates Akt signalling pathway. OPN binds directly to CD44 on the surface of fibroblasts, thereby activating fibroblasts.

There is evidence that TECs release more exosomes in models of unilateral ureteral obstruction, renal ischaemia–reperfusion injury (RIRI), or 5/6 nephrectomy. The use of TGF‐β1 to induce proximal tubular cells y can also make the cells secrete more exosomes, and fibroblast proliferation and activation are observed when these exosomes are cultured with fibroblasts. In addition, the injection of exosomes secreted by damaged TECs into healthy mice through the tail vein induced renal interstitial fibrosis.^110^ In 2021, we developed mouse models of unilateral ureteral obstruction and renal ischaemia–reperfusion. These models were used to investigate the precise mechanism by which exosomes released by injured TECs facilitated the proliferation and activation of renal interstitial fibroblasts. Additionally, we successfully identified the specific microRNAs within these exosomes that were responsible for regulating the activation of fibroblasts. In particular, exosomal miR‐21 derived from TECs could selectively interact with phosphatase and tensin homologue (PTEN), thereby facilitating the proliferation and activation of renal interstitial fibroblasts through the PTEN/AKT signalling pathway. Additionally, exosomal miR‐150‐5p derived from tubular cells promotes fibroblast activation subsequent to RIRI by specifically targeting SOCS‐1.^111^, ^112^ Similarly, injured TECs promote renal fibrosis by delivering exosomes containing miR‐150 to activate fibroblasts.^113^ Chen et al. discovered that exosomes produced by TECs could encapsulate the N‐terminal fragment of osteopontin (N‐OPN) and transport it to fibroblasts. This process promoted fibroblast proliferation and activation via the binding of the encapsulated OPN fragment to CD44.^114^ In summary, damaged TECs can communicate with fibroblasts via exosomes. These reports provide new insights into the mechanism of renal fibrosis and may provide new ideas for the subsequent development of targeted therapies for renal fibrosis (Figure 7).

### 4.6 Exosomes regulate renal fibrosis through other pathways

Changes in cellular metabolism are often accompanied by phenotypic changes. Injury often induces metabolic reprogramming in cells. After kidney injury, TECs and fibroblasts receive a series of signals to induce metabolic reprogramming. Some signals can shift cell metabolism towards glycolysis to satisfy the high energy consumption and biosynthesis demands of damaged cells.^115^, ^116^ PFKFB3 and other pathways that mediate glycolysis promote fibroblast activation and subsequent renal fibrosis, and inhibiting glycolysis in both fibroblasts and TECs can inhibit renal interstitial fibrosis.^117^, ^118^, ^119^ Xu et al. in 2022 showed that exosomes derived from MSCs ameliorated renal fibrosis induced by unilateral ureteral obstruction by inhibiting glycolysis in TECs. Mechanistically, exosomal miR‐21a‐5p decreased glycolysis in TECs by inhibiting the expression of phosphofructokinase muscle isomer, a glycolytic rate‐limiting enzyme.^120^ This work contributed new insights into the mechanism by which exosomes alter cellular metabolism to regulate renal fibrosis.

Mesangial cells are endogenous cells found inside the glomerulus that are situated within the many capillary loops that round the glomerulus. The filtration structure of the glomerular basement membrane is composed of mesangial cells and the mesangial matrix. Mesangial cells have rich biological functions, such as supporting capillaries, secreting matrix, and forming mesangial channels to transport substances. Mesangial cells can be activated under certain pathological conditions, secrete excessive matrix, and cause renal fibrosis.^121^, ^122^ Exosomes may mediate communication between glomerular endothelial cells and mesangial cells. Glomerular endothelial cells treated with high glucose release more exosomes that are enriched in TGF‐β mRNA, which are transported to mesangial cells, induce the activation of mesangial cells and release of extracellular matrix, and promote renal fibrosis.^123^ Furthermore, exosomes originating from MSCs were shown to suppress the proliferation of mesangial cells by modulating the PI3K/Akt and MAPK signalling pathways. Additionally, these exosomes could enhance the expression of the matrix metalloproteinases MMP2 and MMP9, resulting in a reduction in fibronectin and collagen deposition and ultimately improving the condition of renal fibrosis.^124^

Posttranslational modifications can affect the characteristics of some biological macromolecules, regulate signal transduction pathways, and participate in a variety of pathophysiological processes. Core fucosylation is a unique protein modification that is involved in fibrotic processes in a variety of organs.^125^ In the renal interstitial fibrosis model, core fucosylation is an important posttranslational modification of TGF‐β1 and other receptors that can regulate the activation of TGF‐β1 and other profibrotic signalling pathways. Inhibiting core fucosylation can significantly reduce renal pathological injury, fibrosis, and podocyte injury.^126^, ^127^ In 2022, Xu et al. showed that MSC‐derived exosomal miR‐34c‐5p could inhibit the core fucosylation of multiple proteins in macrophages, pericytes and fibroblasts and inhibit the activation of pericytes, macrophages and fibroblasts, thereby improving renal fibrosis.^128^ Thus, we have summarized most of the mechanisms by which exosomes regulate renal fibrosis. As a summary, we summarize in Table 1 the various mechanisms by which exosomes modulate renal fibrosis.

**TABLE 1:** The mechanisms by which exosomes regulate renal fibrosis.

Source of the exosome	Content	Pathways or Mechanisms	Effect on renal fibrosis	References
By regulating EMT				
MSCs	miR‐186‐5p	Reducing EMT in TECs by targeting Smad5	Remission	^59^
Multiple myeloma cells	miR‐21‐5p	Enhancing EMT by targeting TGF‐β/Smad7 signalling pathway	Promotion	^62^
By regulating EndMT				
High glucose‐treated glomerular endothelial cells	TGF‐β1 mRNA	Activating TGF‐β1‐mediated EndMT	Promotion	^76^
Glomerular endothelial cells of AMR patients	Some miRNAs	‐	Promotion	^77^
By regulating autophagy				
MSCs	‐	Inhibiting the mTOR pathway, promoting TECs autophagy	Remission	^91^
Circulatory system	miR‐1‐3p	Targeting ATG13, activating the AKT signalling pathway and inhibiting TECs autophagy	Remission	^92^
MSCs	miR‐122a	Inhibiting autophagy	Remission	^93^
By regulating macrophage polarization				
MSCs	‐	Activating EP2 receptors, inhibiting M1 macrophage polarization	Remission	^101^
MSCs	‐	Promoting M2 macrophage polarization	Remission	^94^, ^102^
TECs	miR‐19b‐3p	Targeting SOCS‐1 to enhance NF‐Κb activity, promoting the activation of M1 macrophages	Promotion	^104^
By activating fibroblasts				
TECs	miR‐21	Targeting PTEN, activating the Akt pathway	Promotion	^111^
TECs	miR‐150‐5p	Targeting and inhibiting SOCS‐1	Promotion	^112^
TECs	miR‐150	‐	Promotion	^113^
TECs	N‐OPN	Binding to CD44	Promotion	^114^
Through other pathways				
Glomerular endothelial cells treated with high glucose	TGF‐β mRNA	Inducing the activation of mesangial cells and release of extracellular matrix	Promotion	^123^
MSCs	‐	Reducing myofibroblast transdifferentiation and cell proliferation and upregulating MMPs in mesangial cells	Remission	^124^
MSCs	miR‐34c‐5p	Inhibiting the core fucosylation of multiple proteins in macrophages, pericytes and fibroblasts	Remission	^128^

## 5 EXOSOMES IN BLOOD AND URINE CAN ACT AS BIOMARKERS FOR RENAL FIBROSIS

Given that exosomes regulate renal fibrosis through a variety of pathways and are released by a variety of cells during renal fibrosis, we believe that exosomes in various body fluids may have great potential as biomarkers to guide the diagnosis and treatment of renal fibrosis. In the past, many mRNAs and miRNAs have been identified in urinary exosomes and peripheral blood exosomes. In recent years, circRNAs, which are a class of long noncoding RNAs with covalently closed loops, have been detected in exosomes.^129^, ^130^ In addition, the N‐OPN and transglutaminase‐2, have been shown to be upregulated in patients with renal fibrosis and in animal models and have the potential to be biomarkers of renal fibrosis.^114^, ^131^ In addition to proteins, a number of exosomes containing certain specific mRNAs, miRNAs, and circRNAs have the potential to become new tools for the management of renal fibrosis (Table 2). Of note, one study examined plasma exosomes. Patients with a high degree of renal fibrosis had high levels of plasma exosomal miRNA‐21. Plasma exosomal miRNA‐21 is expected to be an important biomarker of severe fibrosis after renal transplantation and is conducive to non‐invasive examination.^132^ In addition, numerous studies have shown that urine exosomal miRNA‐29c is a significant biomarker of renal fibrosis, and it exhibits a negative correlation with the extent of renal fibrosis.^57^, ^133^, ^134^, ^135^ Compared to conventional urine and blood biomarkers of renal fibrosis, exosomes may possess greater stability as prospective biomarkers. In conclusion, the use of plasma and urine exosomes as potential indicators of renal fibrosis represents a promising avenue for research. These biomarkers have the potential to provide valuable insights into the diagnosis and treatment of renal fibrosis, presenting distinct benefits over invasive testing methods.

**TABLE 2:** The exosomes and their contents that are biomarkers of renal fibrosis in body fluids.

Type of exosome	Content	Correlation between the expression level and renal fibrosis	Source of the exosome	Reference
Urinary exosome	N‐terminal fragment of osteopontin (N‐OPN)	Positive correlation	TECs	^114^
Urinary exosome	Transglutaminase‐2 (TG2)	Positive correlation	TECs	^131^
Plasma exosome	miRNA‐21	Positive correlation	‐	^132^
Urinary exosome	miRNA‐29c	Negative correlation	‐	^57^, ^133^, ^134^, ^135^
Urinary exosome	miRNA‐200b	Negative correlation	Nonproximal renal tubule	^136^
Urinary exosome	circRNA_0036649	Negative correlation	‐	^137^
Urinary exosome	circRNA_0008925	Positive correlation	TECs	^138^
Urinary exosome	CD2AP mRNA	Negative correlation	Podocytes	^139^
Urinary exosome	miRNA‐150	Positive correlation	‐	^140^
Urinary exosome	miRNA‐451‐5p	Positive correlation	‐	^141^
Urinary exosome	miR‐146a	Negative correlation	‐	^142^
Urinary exosome	miR‐223	Positive correlation	‐	^143^

## 6 THERE IS POTENTIAL TO TRANSLATE EXOSOME RESEARCH INTO THERAPEUTICS FOR RENAL FIBROSIS

The study of exosomes will explore their potential in translational medicine and provide new ideas and ways for creating effective clinical treatment strategies. Naturally secreted exosomes, such as MSCs‐derived exosomes, have therapeutic potential for a variety of diseases.^144^ Besides, an increasing number of novel exosome‐targeted therapeutic delivery systems have been developed. For example, exosome‐mediated delivery of some miRNAs or protein complexes can treat diseases such as liver disease and heart disease.^145^, ^146^ At present, the research on exosomes is more in‐depth, and many engineered exosomes can be used as a therapeutic approach for diseases.^147^ In addition, regulating the biogenesis, secretion, and uptake of exosomes has the potential to increase their beneficial effects on the occurrence and development of diseases or prevent their harmful effects. Understanding the pivotal role of exosomes in renal fibrosis offers substantial therapeutic promise. Converting exosome research into therapies for renal fibrosis are needed.

### 6.1 Naturally secreted exosomes, such as MSCs‐derived exosomes, have therapeutic potential for renal fibrosis

**FIGURE 8:**
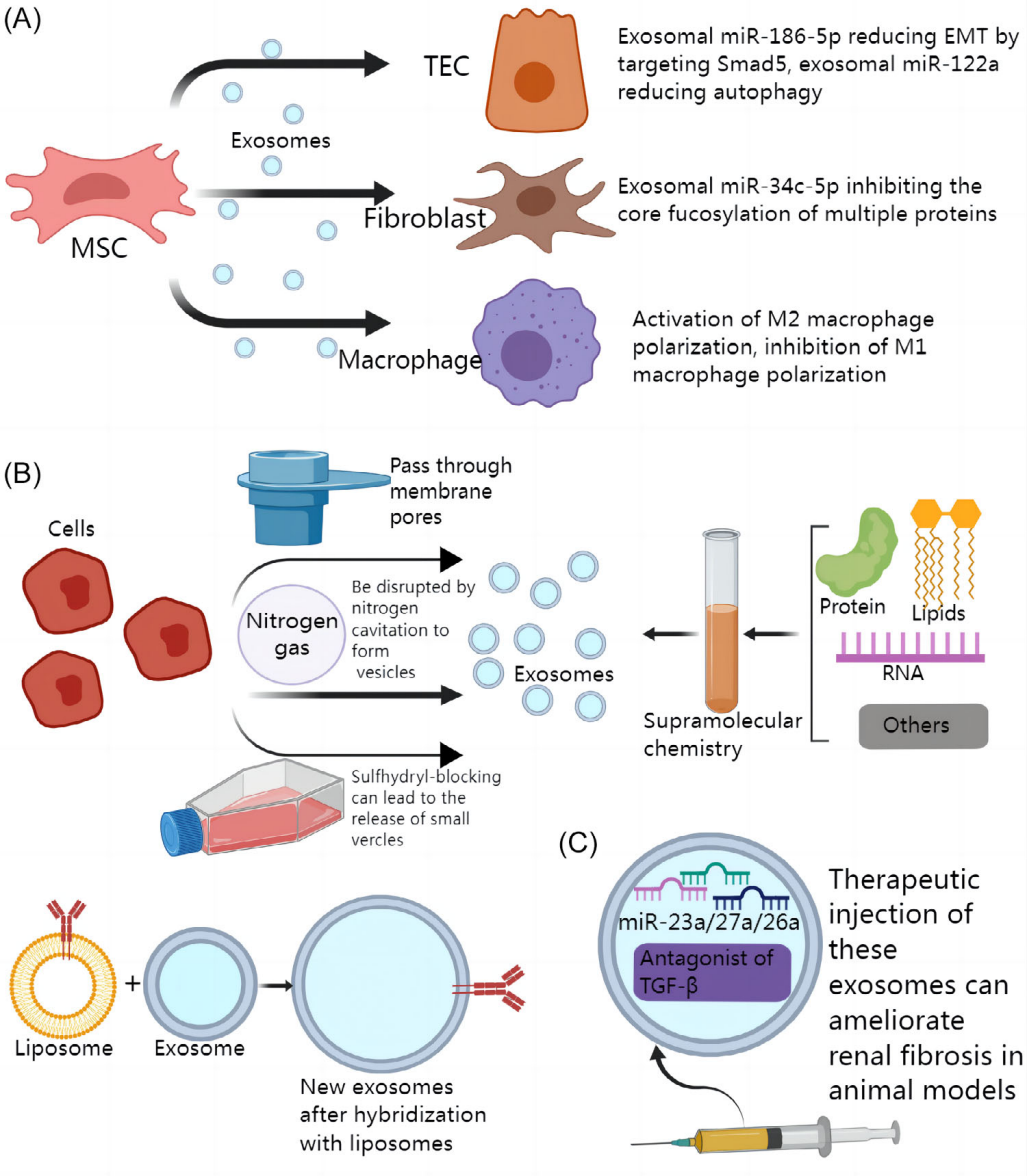
Exosomes for the treatment of renal fibrosis. (A) Mesenchymal stem cells (MSCs)‐derived exosomes have therapeutic potential for renal fibrosis. MSCs‐derived exosomal miR‐186‐5p reduces epithelial–mesenchymal transition (EMT) in tubular epithelial cells (TECs) by targeting Smad5. MSCs‐derived exosomal miR‐34c‐5p inhibits the core fucolation of multiple proteins. MSCs‐derived exosomes can alleviate renal fibrosis by inhibiting the polarization of M1 macrophages and promoting the polarization of M2 macrophages. (B) Fabrication of artificial engineered exosomes. Cells can be forced to pass through membrane pores to form artificial exosomes mimics. Cells can be disrupted by nitrogen cavitation to form artificial exosomes mimics. Key molecules such as lipids and proteins can be synthesized into artificial exosome mimics using supramolecular biochemical techniques. Hybridization of the exosome with the liposome carrying the key molecule can synthesize a new exosome carrying the molecule. (C) Studies have shown that injection of exosomes carrying miR‐23a/27a/26a or TGF‐β antagonists can alleviate renal fibrosis in animal models.

Current strategies for the treatment of renal fibrosis using natural exosomes mainly focus on MSCs. MSCs play a pivotal role in regenerative medicine and biotherapy. MSCs are unique cells with the ability of self‐renewal and multi‐directional differentiation. They have abundant sources and can be isolated from the stroma of almost all organs, and play a pivotal role in regenerative medicine and biotherapy.^148^, ^149^ Previous studies have shown that MSCs can regulate the proliferation, differentiation, migration and metabolic function of parenchymal cells through endocrine or paracrine, limiting the progression of a variety of diseases and improving the prognosis.^150^ Evidence has shown that the therapeutic effects of MSCs on bone diseases, inflammation, and neurodegenerative diseases can be partially attributed to MSC‐derived exosomes.^40^, ^151^, ^152^ As shown in Table 1 and Figure 8, exosomes derived from MSCS can improve renal fibrosis by transporting molecular cargo such as miR‐186‐5p, miR‐122a, and miR‐34c‐5p to target cells such as TECs, macrophages, and fibroblasts (Figure 8A). In addition, the extraction methods of MSCs are increasingly improved, which helps to overcome the challenges of low yield of primary cells, long in vitro expansion time, and reduced differentiation ability after passage.^153^ So we hold the view that therapeutic delivery of MSCs‐derived exosomes and adjustment of the expression level of functional cargo in MSCs‐derived exosomes has great potential for the treatment of renal fibrosis.

### 6.2 Engineered exosomes can be used to treat renal fibrosis

Exosomes are natural carriers of various functional RNAs and proteins, which have the potential to deliver miRNAs, proteins and even small molecule targeted drugs to treat diseases. However, naturally secreted exosomes do not necessarily contain the molecular cargo needed to treat diseases, so using exosomes to load exogenous molecular cargo may be a good solution.^144^, ^154^, ^155^ In this way of thinking, skeletal muscle satellite cell‐derived exosomes carrying miR‐23a/27a/26a cluster were recently constructed by Ji et al. Intravenous administration of these exosomes significantly improved diabetic nephropathy and alleviated renal fibrosis in mice.^156^ In addition, it has been shown that intramuscular injection of miR‐26a‐rich exosomes can directly inhibit the expression of profibrotic protein connective tissue growth factor (CTGF) to alleviate renal fibrosis in mice.^157^ Besides, therapeutic delivery of exosomal miR‐26a can alleviate aldosterone‐induced renal fibrosis by inhibiting CTGF/SMAD3 signalling pathway.^158^ In 2022, Kim et al. successfully introduced an endogenous nanosized antagonist of TGF‐β into cells through the endosomal route. This antagonist was then released into active exosomes, leading to the inhibition of renal fibrosis in animal models. The mechanism of action included the suppression of EMT in TECs^159^ (Figure 8B). Considering the ability of exosomes to efficiently deliver contents to target cells, artificially constructed exosomes rich in specific miRNAs or proteins that regulate fibrosis‐related pathways theoretically have the potential to treat renal fibrosis.

There are still many challenges that limit the clinical development of exosomes in the treatment of renal fibrosis. For example, it is difficult to deliver exosomes to target tissues accurately to exert therapeutic effects.^147^ To overcome these challenges, the development of targeted exosomes is critical. Some researchers have suggested that the targeting of exosomes can be enhanced by exosome–liposome hybridization, using bioengineering techniques to modify exosome receptor and ligand proteins, and so on.^160^, ^161^ For example, exosomes can be targeted to specific cells by modifying transmembrane protein signal peptides on the surface of exosomes to display ligands on the external surface of exosomes.^162^ In 2023, Guo et al. used exosomes inserted into Golgi glycoprotein 1 to transport Wnt agonist 1 to target inhibition of osteoblastic bone formation and bone loss.^163^ It is a pity that there are no examples of studies using targeted exosomes for the treatment of renal fibrosis. But we believe that new bioengineering technology can be rationally used to develop exosomes targeting target cells such as renal fibroblasts, renal TECs, macrophages, and glomerular endothelial cells for interfering with the pathophysiological process of renal fibrosis.

All of the above are based on naturally occurring exosomes as well as anthropogenic partial surface modification or limited alteration of their contents. There are many difficulties in the purification, storage, drug delivery, and quality control of natural exosomest.^164^ The emergence of artificial exosomes based on nanobiotic technology helps to overcome the current challenges and has the potential to be clinically transformed into the treatment of renal fibrosis. Specifically, the current techniques for constructing artificial exosomes can be divided into three categories. One is to decompose relatively large and complex cells into small vesicles with simple components.^165^ Second, lipids, proteins and other components are biochemically fused into vesicles to mimic the structure and function of exosomes.^166^ Third, liposomes conjugated with specific antibodies, inserted ligands on the membrane surface, or combined with special functional components can be fused and hybridized with exosomes to form new functional engineered exosomes^165^, ^167^, ^168^, ^169^ (Figure 8C). Although there is still a lack of research on the treatment of renal fibrosis with artificial engineered exosomes, we believe that this direction has a good prospect.

### 6.3 Modulation of exosome biosynthesis, release, and uptake also has the potential to be a therapeutic option for renal fibrosis

We believe that regulating the biosynthesis, release and uptake of some exosomes also has the potential to innovate the treatment of renal fibrosis. For example, Sinomenine can delay the progression of renal fibrosis in mice with ureteral obstruction by promoting the secretion of exosomes derived from bone marrow MSCs.^170^ It is worth noting that in diabetic nephropathy model, HNRNPA1 mediated exosome sorting could transport miR‐483‐5p from TECs to urine, thereby alleviating the inhibition of MAPK1 and TIMP2 mRNA by intracellular miR‐483‐5p and relieving the anti‐fibrosis effect mediated by miR‐483‐5p.^171^ Interestingly, Xu et al. recently found that Gan‐song Yin, a traditional Chinese medicine, can interfering with the secretion of exosomes by adipocytes and affecting the level of miR‐21‐5p in exosomes, thus promoting miR‐21‐5p to targeting inhibit TGF‐β1 and alleviate renal fibrosis caused by diabetic nephropathy.^172^ Similarly, another traditional Chinese medicine, Huangqi Chifeng decoction, can improve renal fibrosis caused by IgA nephropathy through TGF‐β1/Smad3 signalling by inhibiting the secretion of exosomes containing TGF‐β1 mRNA.^173^ Therefore, delving deeper into therapeutic approaches centred on regulating exosomes or related components to combat kidney fibrosis can chart a course for future research and clinical advancements. While the majority of existing research on the treatment of renal fibrosis with exosomes has mostly been conducted using cell and animal models, exosomes serve as a fundamental framework that offers valuable insights into potential future treatments targeting renal fibrosis.

## 7 SUMMARY AND DISCUSSION

Exosomes are involved in the transport of materials, the transduction of signals, and the transmission of information among cells by conveying and transferring diverse molecular mediators. Exosomes play a crucial role in the intricate physiological and pathological regulation of the kidney. They facilitate communication between various cell types, including TECs, macrophages, fibroblasts, podocytes, and mesangial cells. Moreover, exosomes are responsible for regulating key processes within the kidney, such as the renal inflammatory response, injury repair, and the immune response. Currently, there is significant interest in the role of exosomes in normal kidney function and a range of renal disorders. Exosomes have been shown to possess distinctive functions in the progression of renal fibrosis induced by diverse causes; nevertheless, a comprehensive understanding of their underlying mechanism remains incomplete.

This review focused on the involvement of exosomes in the modulation of renal fibrosis and the advancements made in related research. The following traits were identified. Exosomes play a crucial role in facilitating intercellular communication among various cell types within the renal system, including TECs and fibroblasts, glomerular endothelial cells and mesangial cells, and TECs and macrophages. Through their regulatory functions, exosomes modulate renal fibrosis by influencing processes such as epithelial mesenchymal transformation, autophagy, macrophage polarization, fibroblast activation, glycolysis, and mesangial cell activation. The regulatory mechanisms of exosomes in renal fibrosis are intricate, and there are potential interactions and reciprocal regulation among EMT, fibroblast activation, and macrophage polarization. Hence, the comprehensive elucidation of the mechanism by which exosomes regulate renal fibrosis is a challenging task. Besides, exosomes play a significant role in the pathogenesis of renal fibrosis and serve as a valuable reservoir of potential biomarkers for this condition.

Many studies have been conducted on the use of exosomes as therapeutic agents for the treatment of various disorders. The field of stem cell therapy is continuously advancing, and there is growing interest in using exosomes produced by MSCs for the treatment of renal fibrosis. Moreover, existing methodologies have been used to synthetically engineer exosomes and facilitate their uptake by certain cells, thus enabling their intended functionality. Urinary and plasma exosomes can serve as reservoirs of biomarkers of renal fibrosis, thereby facilitating the identification and management of this pathological condition. It is believed that with the in‐depth study of the relationship between exosomes and renal fibrosis and the improvement of clinical research, there will be an increasing number of targeted diagnosis and treatment methods for renal fibrosis in the future.

## AUTHOR CONTRIBUTIONS

Peihan Wang wrote, revised, and edited the manuscript, made the figures and tables. Wu Chen, Bojun Li, Songyuan Yang, Wei Li, Sheng Zhao, Jinzhuo Ning, and Xiangjun Zhou reviewed this manuscript. Fan Cheng conceptualized and supervised the work. Peihan Wang and Wu Chen contributed equally. Fan Cheng is the corresponding author. The final manuscript was reviewed and approved by all authors.

## CONFLICT OF INTEREST STATEMENT

The authors declare that there was no competing interests.
